# Oroxylin A shows limited antiviral activity towards dengue virus

**DOI:** 10.1186/s13104-022-06040-0

**Published:** 2022-05-04

**Authors:** Thippayawan Ratanakomol, Sittiruk Roytrakul, Nitwara Wikan, Duncan R. Smith

**Affiliations:** 1 Institute of Molecular Biosciences, Mahidol University, Salaya, 73170 Thailand; 2 National Center for Genetic Engineering and Biotechnology (BIOTEC), National Science and Technology Development Agency, Pathum Thani, 12120 Thailand

**Keywords:** Oroxylin A, Dengue virus, Antiviral compound

## Abstract

**Objective:**

The mosquito transmitted dengue virus (DENV) the causative agent of dengue fever (DF) remains a significant public health burden in many countries. Thailand, along with many countries in Asia and elsewhere, has a long history of using traditional medicines to combat febrile diseases such as DF. Screening bioactive compounds from traditional medicines reported to have antipyretic or anti-inflammatory activity may lead to the development of potent antivirals. In this study oroxylin A (OA), a flavonoid derivative found in *Oroxylum indicum* (commonly called the Indian trumpet flower or tree of Damocles), was screened for antiviral activity towards DENV.

**Results:**

Cytotoxicity analysis in BHK-21 cells showed a 50% cytotoxic concentration (CC_50_) of 534.17 µM. The compound showed no direct virucidal activity towards DENV, and pre-treatment of cells had no effect on virus production. A deficit was seen in virus production when cells were post-infection treated with oroxylin A. Under conditions of post-infection treatment, the EC_50_ value was 201.1 µM, giving a selectivity index (SI) value of 2.66. Accumulation of DENV E protein inside the cell was seen under conditions of post-infection treatment, suggesting that oroxylin A may exert some effects at the virus assembly/egress stages of the replication cycle.

**Supplementary Information:**

The online version contains supplementary material available at 10.1186/s13104-022-06040-0.

## Introduction

The mosquito transmitted dengue virus (DENV) is the most prevalent arthropod transmitted virus in the world causing some 390 million cases of human infection per year, of which approximately one-quarter result in some degree of symptomatic presentation [1]. Where symptomatic, infection can result in a wide range of presentation from an essentially mild self-limiting febrile disease to a severe life threatening disease primarily characterized by hemorrhage and the resultant complications [2]. DENV is believed to have emerged from a sylvatic cycle with the primary hosts being non-human primates to a human epidemic/endemic cycle some 1000–1500 years ago [3]. Since its emergence into the human population treatment for DENV will primarily have been through the use of natural medicines which were believed to have anti-inflammatory or antipyretic activity [4], and thus it is possible that some plants used traditionally to treat febrile diseases contain bioactive constituents with antiviral activity.

Thailand, as with many countries in Asia has a long history of natural medicine usage, with a number of plants being identified as having antipyretic properties. Based on the identification of compounds from plants that have antipyretic use in Thai medicine we have previously shown that the flavonoid kaempferol, a constituent of *Moringa oleifera* and *Coccinia grandis* amongst a number of other sources, berberine a bioactive constituent of *Tinospora crispa* and *Berberis vulgaris* and andrographolide from *Andrographis paniculata* possess anti-flaviviral activities [5–7]. As part of our ongoing work in identifying antiviral agents from Thai natural medicines we screened oroxylin A (OA), an O-methylated flavone that is found in several parts of *Oroxylum indicum* [8]. *O. indicum* (commonly called the Indian trumpet flower or tree of Damocles, and “Phaekaa” in Thai) has been identified as having antipyretic activities, and studies on a bioactive constituent, oroxylin A have suggested that it possesses anti-cancer, anti-inflammatory, neuroprotective and proapoptotic activities [8]. In addition OA has also been shown to possess broad anti-viral activity against Coxsackievirus B3 [9], respiratory syncytial virus [10], influenza virus [11] as well as to have a cytoprotective effect against enterovirus 71 [12]. Thus, OA was seen as a candidate compound worth evaluating for activity against DENV.

## Main text

### Materials and methods

#### Cell culture and virus

BHK-21 (ATCC No. CCL-10), African green monkey kidney cell line Vero (ATCC No. CCL-81) and the Rhesus monkey kidney epithelial cell line LLC-MK2 (ATCC No. CCL-7) were cultured in either 10% or 5% fetal bovine serum (FBS, Thermo Fisher Scientific, Waltham, MA) in Dulbecco’s modified Eagle’s medium (DMEM; Gibco BRL, Gaithersburg, MD) with 100 units/ml of penicillin/streptomycin solution (Pen/Strep, Merck KGaA, Darmstadt, Germany) and maintained at 37 °C with 5% CO_2_. C6/36 cells (ATCC CRL-1660) were cultured at 28 °C in 10% FBS in minimum essential medium (MEM, Thermo Fisher Scientific, Waltham, MA) with ambient CO_2_.

DENV 2 (strain 16681) was propagated in C6/36 cells as described previously [13]. Virus titer was determined by plaque assay on LLC-MK_2_ cells as described previously [13].

#### Compound preparation

OA (Additional file 1: Fig. 1) (CAS no. 480115; Chengdu Biopurify Phytochemicals Ltd., Sichuan, China) was dissolved in absolute dimethyl sulfoxide (DMSO, Sigma-Aldrich, St. Louis, MO). DMSO was also used as a diluent control. Desired concentrations were obtained by further diluting in culture media from an original stock of 100 mM.

**Fig. 1:**
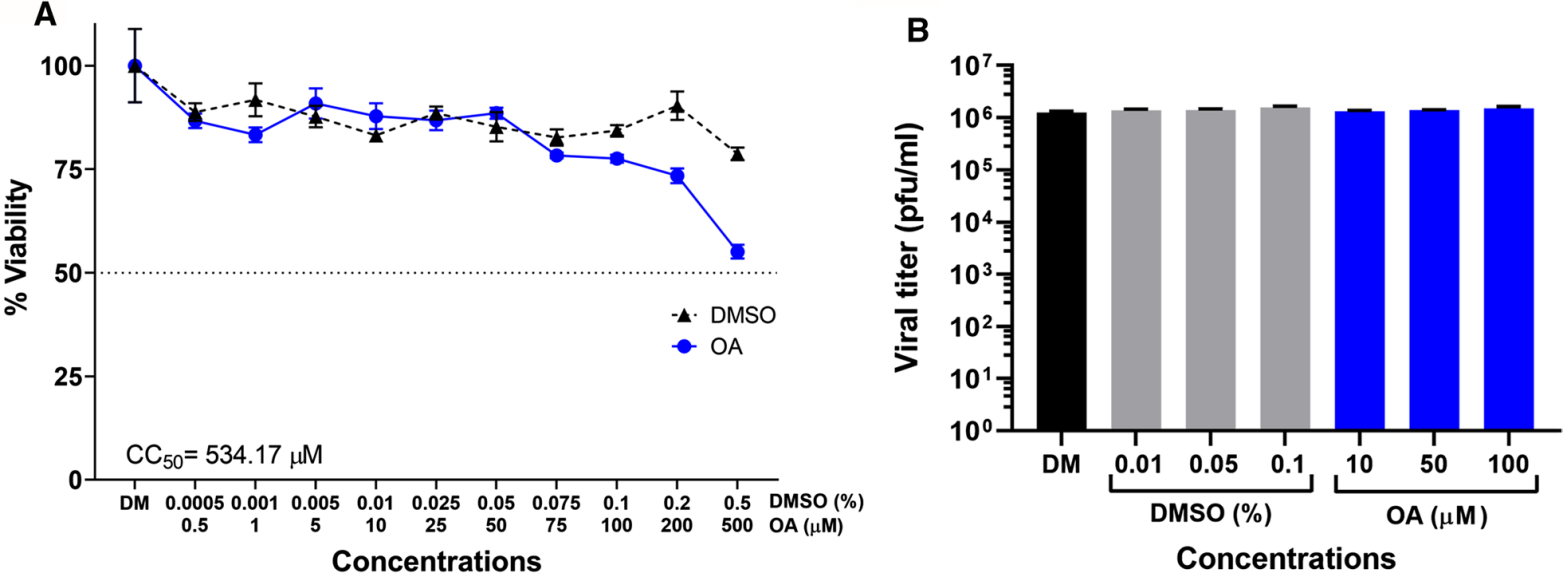
Cytotoxicity and virucidal activity of oroxylin A. **A** Cell viability of BHK-21 cells after 24 h incubation with OA or vehicle control was assessed by the MTT assay and data was used to calculate the CC_50_. **B** Virucidal activity of OA towards DENV was assessed by incubating stock virus with different concentrations of OA or equivalent vehicle control (DMSO) for 1 h, after which virus titer was determined by plaque assay. Data is shown as mean ± S.E.M. Cytotoxicity analysis was undertaken as four independent replicates, while virucidal activity was assessed with three independent biological replicates with duplicate plaque assay

#### Cytotoxicity and virucidal activity

BHK-21 cells were cultured for 18–20 h to achieve 70–80% confluency, after which cells were treated with OA (0.5–500 µM) or DMSO (0.0005–0.5%) for 24 h before assessing cell viability using the thiazolyl blue tetrazolium bromide dye (MTT) assay (Applichem GmBH, Darmstadt, Germany). The intensity of the dissolved formazan was measured at 570 nm (Beckman Coulter DX880 ST-52, Brea, CA). Experiment was undertaken as four independent replicates.

Assessment of virucidal activity was undertaken as previously described [5, 7, 14] and was performed by direct incubation of stock DENV 2 with OA (10, 50 or 100 µM) or DMSO (0.01, 0.05, 0.1%) or DMEM for 1 h at 37 °C. Viral titer was then determined by plaque assay as described elsewhere [13]. Experiment was undertaken as three independent replicates, with duplicate plaque assay.

#### Compound activity assays

The treatment conditions were essentially previously described [7]. Briefly BHK-21 cells were treated with selected concentrations of OA (or vehicle control) in three different treatment conditions. In pre-treatment, cells were treated with OA for 2 h prior to DENV 2 (MOI 2) or mock infection and after 2 h incubation cells were washed with PBS and then maintained in complete media under standard conditions for 24 h. In post-treatment, cells were infected with DENV 2 or mock infected for 2 h before washing with PBS and adding complete media containing OA or vehicle control before incubation under standard conditions for 24 h. In the combined pre- and post-infection treatment, cells were both pre-incubated and post infection incubated with compound or vehicle control, but the infection was undertaken in the absence of the compound or vehicle control. At 24 h post-infection, cell pellets were collected for flow cytometry analysis of viral infectivity as previously described [7], and the supernatants of the same conditions were also collected for standard plaque assays to determine infectious viral titer.

#### Flow cytometry

Flow cytometry was undertaken as previously described [7]. Briefly, cells were collected by trypsinization followed by centrifugation, blocked with 10% goat serum (Thermo Fisher Scientific, Waltham, MA) before fixing with 4% paraformaldehyde (Merck KGaA, Darmstadt, Germany) and subsequently permeabilizing with 0.2% Triton X-100. Cells were incubated with a 1:150 dilution of a pan-specific mouse anti-DENV E protein monoclonal antibody from hybridoma HB114 [15], and subsequently with a 1:40 dilution of a goat anti-mouse IgG conjugated with fluorescein isothiocyanate. Samples were run on a BD FACSCalibur cytometer (Becton Dickinson, BD Biosciences, San Jose, CA), using CELLQuest pro (Version 6.0) software. All experiments were undertaken independently in triplicate.

#### Western blotting

Mock infected or DENV 2 infected cells treated or untreated with OA as appropriate were collected by trypsinization followed by centrifugation at 5000*g* at 4 °C for 5 min and were subsequently lysed with 100 µl of radioimmunoprecipitation (RIPA) buffer containing protease inhibitor cocktail (Bio Basic Inc., Markham, Canada) and kept on ice for 30 min with vortexing every 10 min, and then centrifuged at 12,250*g* at 4 °C for 15 min. Protein concentrations were measured by the Bradford assay. Protein were separated by electrophoresis though 10% SDS polyacrylamide gels before transfer to 0.2 µm nitrocellulose membranes (GE Healthcare, Buckinghamshire, UK). Membranes were subsequently blocked with 5% skim milk in TBS/0.05% Tween 20 at room temperature and subsequently incubated with a pan-specific mouse anti-flavivirus E protein monoclonal antibody from hybridoma HB112 [15] at a 1:500 dilution, or a rabbit anti-dengue type 2 NS1 antibody at a 1:2000 dilution (PA5-32207; Thermo Scientific, Waltham, MA), or a rabbit polyclonal anti-DENV 2 NS3 antibody at a 1:8000 dilution (GTX124252; GeneTex Inc., Irvine, CA), or a mouse anti-DENV 2 NS5 monoclonal antibody at a 1:5000 dilution (MA5-17295, Thermo Fisher Scientific, Waltham, MA), or a 1:5000 dilution of mouse anti-GAPDH monoclonal antibody (sc-32233; Santa Cruz Biotechnology Inc., Dallas, TX) overnight at 4 °C. Secondary antibodies were either a horseradish peroxidase (HRP) conjugated goat anti-mouse IgG at a 1:5000 dilution (A4416, Sigma-Aldrich, St.Louis, MO) or a HRP-conjugated goat anti-rabbit IgG at a 1:8000 dilution (31460, Pierce, Rockford, IL) as appropriate, and these were incubated with the membrane for 1 h at room temperature. The signals were developed with the Amersham ECL plus Western Blotting Detection Reagents (GE Healthcare, Chicago, IL) and immediately captured using a visible western blot imaging system (Azure c400, Azure Biosystems, Inc., Dublin, CA).

#### Data analysis

The CC_50_ estimation was performed using AAT Bioquest-calculator: https://www.aatbio.com/tools/ic50-calculator (accessed on 23 March 2022) with a four-parameter, background correction (subtract smallest response), and normalization (divide by largest response). Statistical analysis was performed with GraphPad Prism 9 for Windows (GraphPad Software Inc., San Diego, CA); EC_50_ was calculated using a non-linear fit dose response curve, multiple sample comparison using one way ANOVA comparing to DMSO control. Data is shown as mean ± S.E.M. Significance is denoted by *p ≤ 0.05, **p ≤ 0.01 ***p ≤ 0.001, ****p ≤ 0.0001.

## Results

### Cytotoxicity profile and virucidal activity

To determine the cytotoxicity of OA, BHK-21 cells were incubated with OA (0.5–500 µM) or equivalent vehicle control (DMSO, 0.0005–0.5%) for 24 h under standard conditions before cell viability was assessed by the MTT assay. The results (Fig. 1A) showed that the half maximal cytotoxicity concentration (CC_50_) of OA towards BHK-21 cell was 534.17 µM. To determine if OA exerts a direct effect on the DENV virion, stock virus was incubated with increasing concentrations of OA (10–100 µM) or vehicle control (DMSO at 0.01–0.1%) for 1 h at 37 °C, before establishing the virus titer by plaque assay. Results (Fig. 1B) showed no loss of titer after incubation with OA, suggesting this compound does not directly affect the DENV virion.

### Compound activity assays

**Fig. 2:**
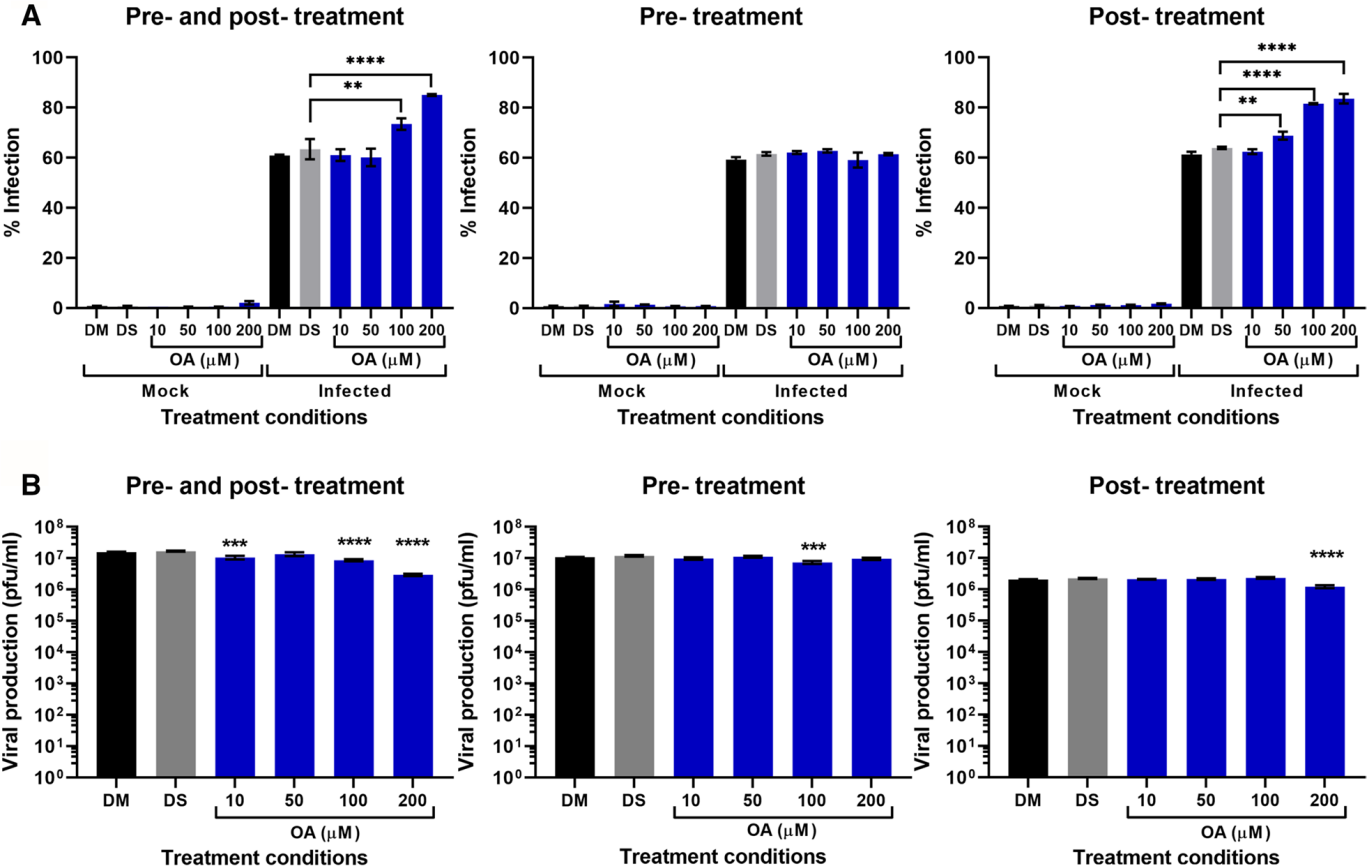
Dengue virus infectivity and productivity in different treatment conditions. BHK-21 cells were treated with OA in three different conditions: pre- and post-treatment, pre-treatment, or post-treatment separately with either mock or DENV 2 infection. **A** Cells from each condition were collected to assess viral infectivity by flow cytometry which detects viral E protein while **B** supernatants from the same conditions were collected and assessed for viral productivity by standard plaque assay. Data is shown as mean ± S.E.M. Significance is denoted by *p ≤ 0.05, **p ≤ 0.01 ***p ≤ 0.001, ****p ≤ 0.0001. Experiments were undertaken as three independent biological replicates, with duplicate plaque assay where appropriate

To determine if OA could exert an effect on DENV through cellular mechanisms, three compound treatment conditions were investigated, pre-treatment infection of cells alone, post infection treatment of cells alone and a combined pre- and post-infection treatment. Analysis included determining the level of infection by flow cytometry and plaque assay to investigate virus titer in the supernatant. Flow cytometry analysis of cells found significant changes in the level of infection in pre- and post-infection treatment at OA concentrations of 100 and 200 µM, and in post-infection treated cells at concentrations above 50 µM (Fig. 2A). Markedly, both conditions showed an increase in levels of infection. No change was seen in cells that were only pre-treated before infection.

New virion production in supernatants was determined by plaque assay. Results showed no effect upon viral titer in cells that were pre-treated before infection only (Fig. 2B). Relatively small reductions in virus titer were seen when cells were treated with OA both pre- and post-infection, and in post-infection only. The EC_50_ for post-infection treatment was 205.7 µM, giving a selectivity index (SI) of 2.66, while the EC_50_ for a combined pre- and post-infection treatment was 102.3 µM, with a selectivity index of 5.28.

**Fig. 3:**
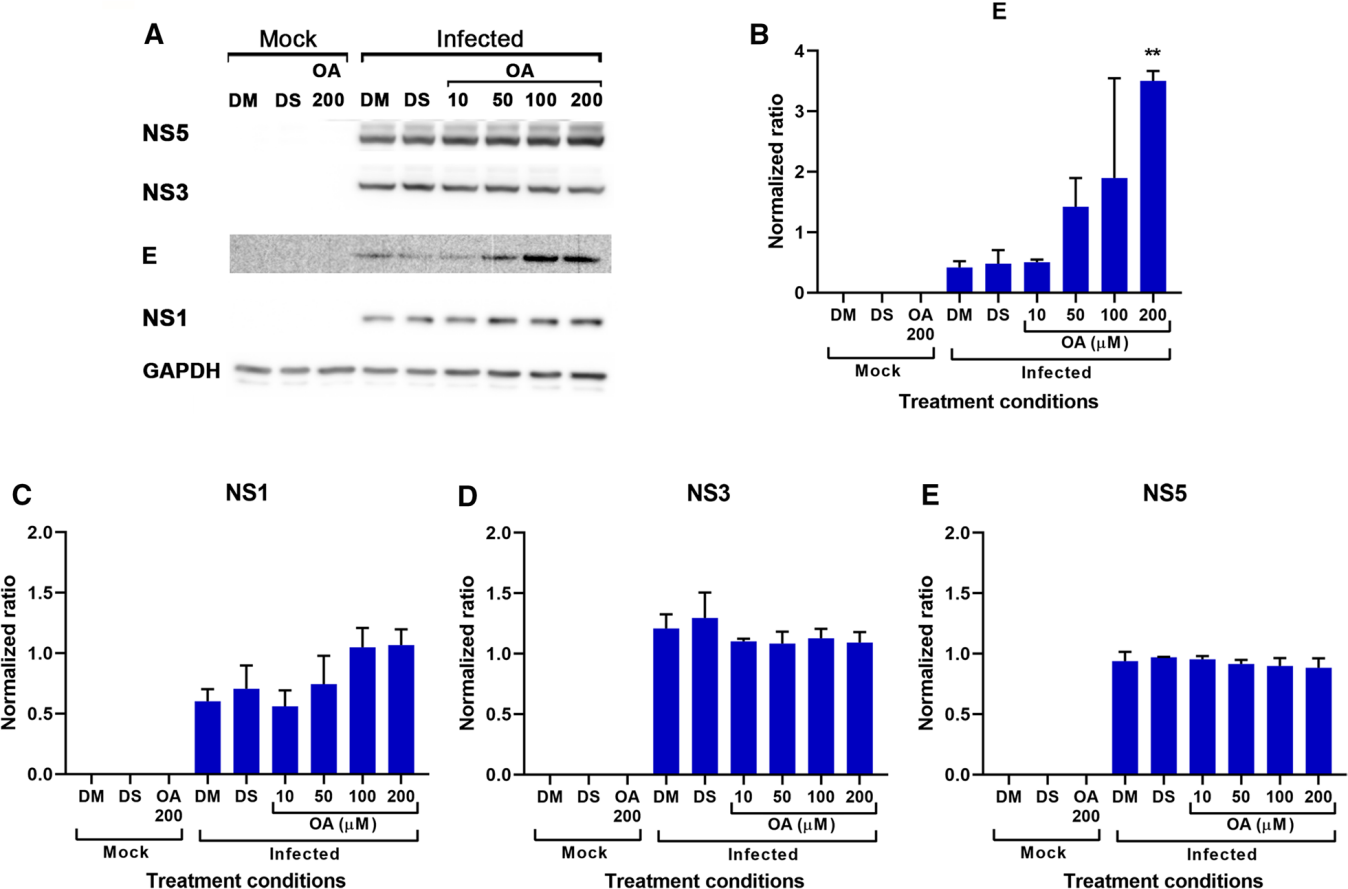
Effect of OA on DENV infection. BHK-21 cells were mock or DENV 2 infected before being treated with an equivalent volume of DMEM (DM), or DMSO solvent control (DS) or various concentrations of OA for 24 h. **A** After 24 h levels of DENV E, NS1, NS3 and NS5 were determined by western blot in parallel with host cell GAPDH as a control. **B**–**E** Band intensities from **A** were quantitated and normalized to expression of GAPDH. All experiments were undertaken independently in triplicate. Error bars show + S.E.M. *p ≤ 0.05, **p ≤ 0.01). Uncropped western blots for all replicates are provided in the associated supplemental materials

The apparent increase in level of infection see under OA treatment would be consistent with the increased accumulation of E protein within the cell under conditions of treatment. We therefore repeated the post-infection treatment experiment and determined the level of expression of four DENV proteins (E, NS1, NS3 and NS5) as well as the expression of GAPDH for normalization by western blotting. The results (Fig. 3) showed a significant, dose dependent increase in E protein expression, but no significant change in expression of NS1, NS3 and NS5 (Fig. 3) was seen. However, there was evidence of a trend of increased NS1 expression, but as noted this did not reach significance.

### Discussion

OA, is an O-methylated flavone that has been shown to have bioactivity in a number of systems [8], and additionally has been reported to possess antiviral activity [9–12]. In this study OA was shown to have some anti-DENV activity, but with a low SI. In this instance the selectivity index indicates the ratio between the compounds toxicity and its effective antiviral ability, and while there is no absolute rule, compounds with an SI of < 10 are generally not considered suitable for further development as an antiviral compound. It is clear however that OA is having some, limited antiviral activity. The reduction of virus titer is associated with an accumulation of E protein, and to a lesser and non-significant extent NS1, suggesting a direct effect on viral production rather than just a non-specific degradation of the cells ability to produce virus. Markedly, both E and NS1 egress from the cell, with E protein being part of the mature virion released from the cell, and NS1 having a secreted form [16]. Thus, the effects are consistent with OA affecting egress/secretion. In a recent study on the isoquinoline alkaloid berberine a similar phenomenon was observed, with berberine increasing apparent infection levels while showing reduced virus output [7], possibly indicating that the two compounds act through a similar mechanism. However, berberine also showed a significant virucidal activity [7] and it is probable that the combined dual action of berberine is what made it a more effective antiviral agent than OA as seen in this study.

### Limitations

This study was conducted using a single virus strain (DENV 2) and a single cell line (BHK21). Our previous studies have shown that both virus strain [17] and cell line [5] can influence the antiviral activity of a compound. However, given the low SI values, especially in post-infection treatment it is difficult to foresee that further investigation of this compound as an anti-DENV antiviral are warranted.

## Supplementary Information

**Additional file 1: Figure S1. **Chemical structure of oroxylin A and uncropped western blots.

## Data Availability

The datasets used and/or analysed during the current study available from the corresponding author on reasonable request.

## Abbreviations

DENV: Dengue virus
OA: Oroxylin A
