# Supplementary material for: Oroxylin A shows limited antiviral activity towards dengue virus
Source: BMC Res Notes. 2022 May 4;15:154. doi: 10.1186/s13104-022-06040-0 (PMC9066930; doi:10.1186/s13104-022-06040-0)
Supplement: Supplementary file 1 — Additional file 1: Figure S1. Chemical structure of oroxylin A and uncropped western blots. [file 13104_2022_6040_MOESM1_ESM.pdf]

Supplemental materials

Oroxylin A shows limited antiviral activity towards dengue virus

Thippayawan Ratanakomol [1], Sittiruk Roytrakul [2], Nitwara Wikan [1], and Duncan R. Smith [1],*

[1]Institute of Molecular Biosciences, Mahidol University, Salaya, 73170, Thailand;

[2]National Center for Genetic Engineering and Biotechnology (BIOTEC), National Science and Technology Development Agency, Pathum Thani, 12120, Thailand;

*Correspondence: duncan_r_smith@hotmail.com; Tel.: +66-2800-3624-8

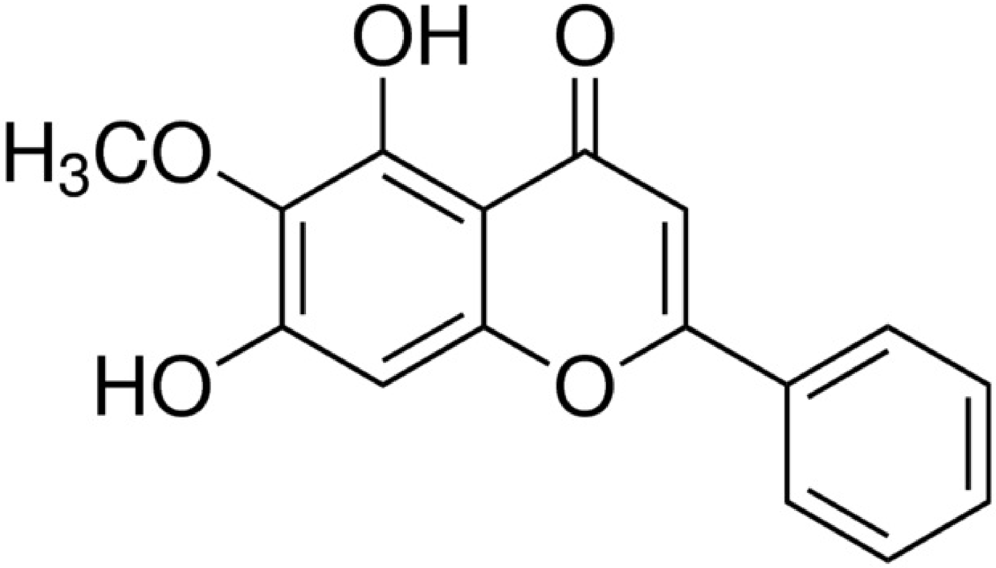

Additional Figure S1. Chemical structure of oroxylin A.

Uncropped western blots

Western blot of DENV 2 protein expression in 24 h oroxylin A post-treatment condition

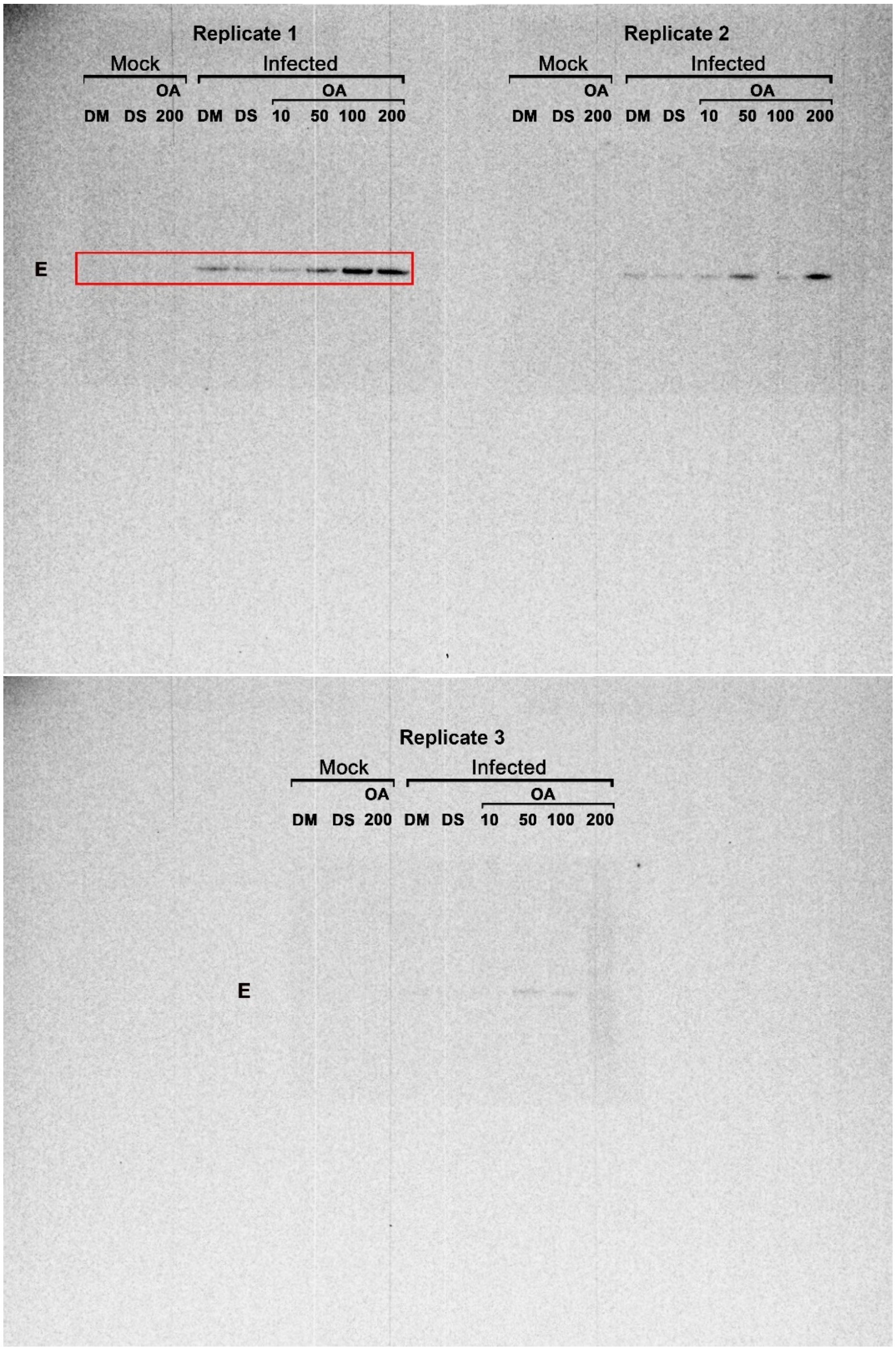

Full western blot membranes of E proteins captured by Azure400 (Replicate 1-3), red box indicates bands presented in Fig 3; E proteins.

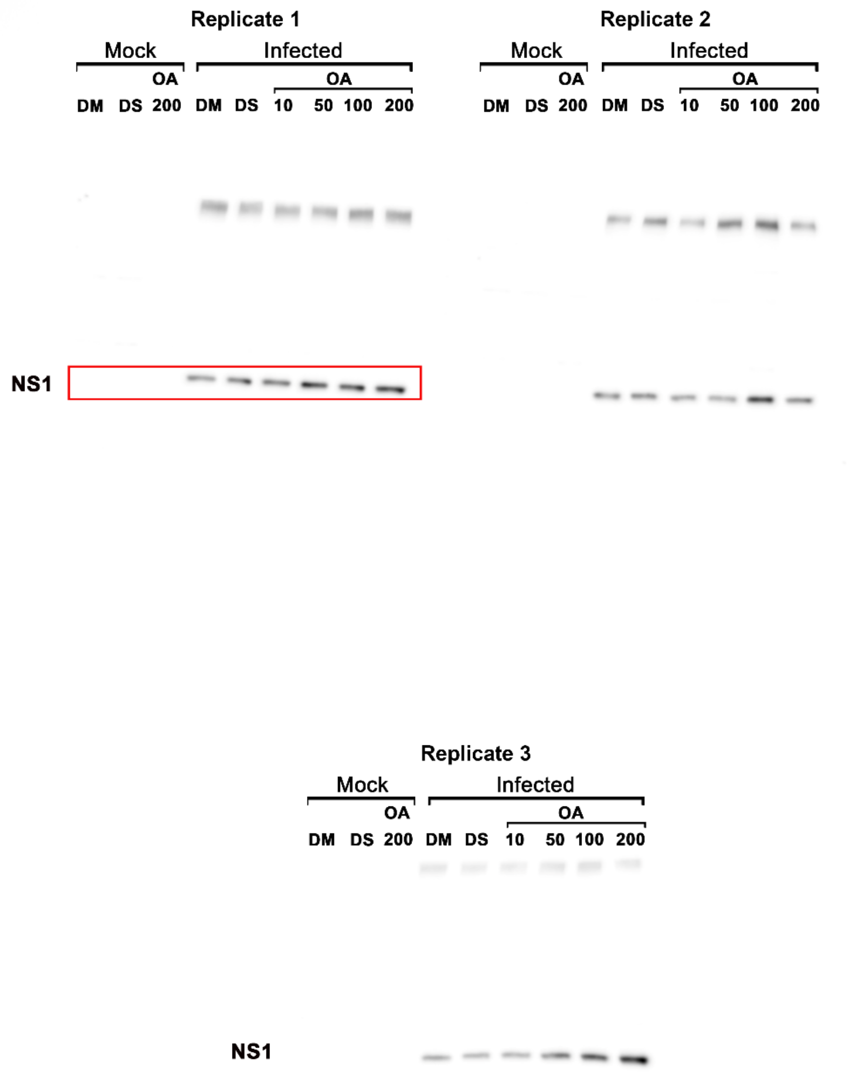

Full western blot membranes of NS1 proteins captured by Azure400 (Replicate 1-3), red box indicates bands presented in Fig 3; NS1 proteins.

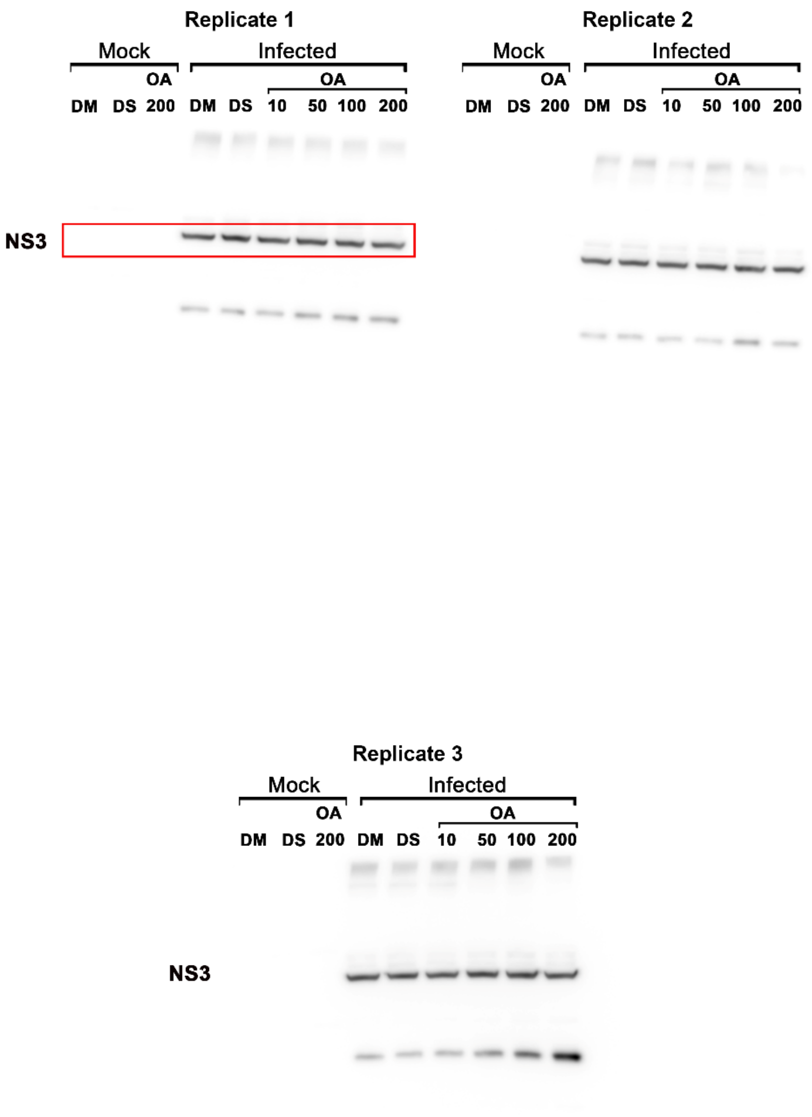

Full western blot membranes of NS3 proteins captured by Azure400 (Replicate 1-3), red box indicates bands presented in Fig 3; NS3 proteins.

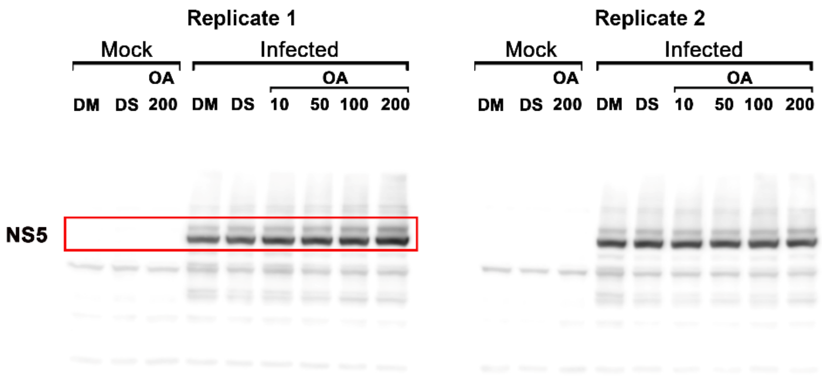

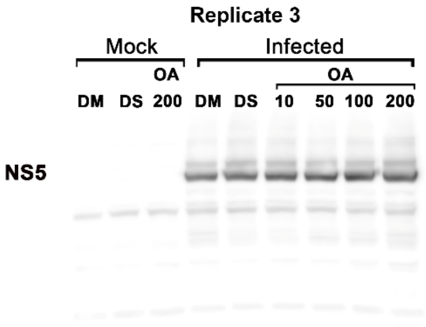

Full western blot membranes of NS5 proteins captured by Azure400 (Replicate 1-3), red box indicates bands presented in Fig 3; NS5 proteins.

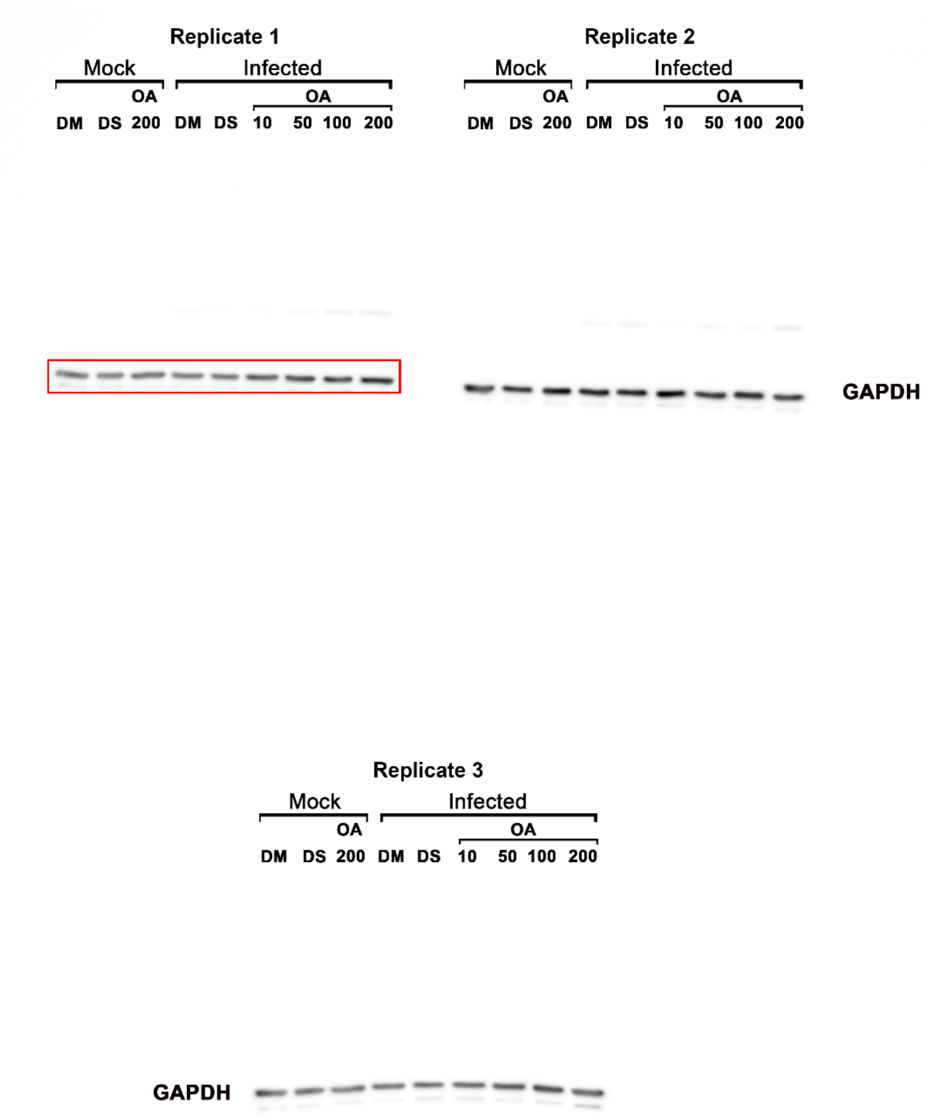

Full western blot membranes of GAPDH (normalized control) captured by Azure400 (Replicate 1-3), red box indicates bands presented in Fig 3; GAPDH.
